# Provider knowledge of treatment policy and dosing regimen with artemether-lumefantrine and quinine in malaria-endemic areas of western Kenya

**DOI:** 10.1186/1475-2875-11-436

**Published:** 2012-12-29

**Authors:** Carren A Watsierah, Rosebella O Onyango, James H Ombaka, Benard O Abong’o, Collins Ouma

**Affiliations:** 1 Department of Public Health, Maseno University, Private Bag, Maseno, Kenya; 2 Department of Biomedical Sciences and Technology, Maseno University, Private Bag, Maseno, Kenya

## Abstract

**Background:**

Due to widespread anti-malarial drug resistance in many countries, Kenya included, artemisinin-based Combination Therapy (ACT) has been adopted as the most effective treatment option against malaria. Artemether-lumefantrine (AL) is the first-line ACT for treatment of uncomplicated malaria in Kenya, while quinine is preferred for complicated and severe malaria. Information on the providers’ knowledge and practices prior to or during AL and quinine implementation is scanty. The current study evaluated providers’ knowledge and practices of treatment policy and dosing regimens with AL and quinine in the public, private and not-for-profit drug outlets.

**Methods:**

A cross-sectional survey using three-stage sampling of 288 (126 public, 96 private and 66 not-for-profits) providers in drug outlets was conducted in western Kenya in two *Plasmodium falciparum*-endemic regions with varying malarial risk. Information on provider in-service training, knowledge (qualification, treatment policy, dosing regimen, recently banned anti-malarials) and on practices (request for written prescription, prescription of AL, selling partial packs and advice given to patients after prescription), was collected.

**Results:**

Only 15.6% of providers in private outlets had received any in-service training on AL use. All (100%) in public and majority (98.4%) in not-for-profit outlets mentioned AL as first line-treatment drug. Quinine was mentioned as second-line drug by 47.9% in private outlets. A total of 92.0% in public, 57.3% in private and 78.8% in not-for-profit outlets stated correct AL dose for adults. A total of 85.7% of providers in public, 30.2% in private and 41.0% in not-for-profit outlets were aware that SP recommendations changed from treatment for mild malaria to IPTp in high risk areas. In-service training influenced treatment regimen for uncomplicated malaria (*P* = 0.039 and *P* = 0.039) and severe malaria (*P* < 0.0001 and *P* = 0.002) in children and adults, respectively. Most (82.3%) of private outlets sell partial packs of AL while 72.4% do not request for written prescription for AL. In-service training influenced request for written prescription (*P* = 0.001), AL prescription (*P* < 0.0001) and selling of partial packs (*P* < 0.0001).

**Conclusion:**

Public-sector providers have higher knowledge on treatment policy and dosing regimen on recommended anti-malarials. Changes in treatment guidelines should be accompanied by subsequent implementation activities involving all sector players in unbiased strategies.

## Background

The burden of malaria persists in many parts of Africa despite the availability of many interventions that are focused on preventive and therapeutic strategies [1]. Artemisinin-based Combination Therapy (ACT) has been adopted as the most effective treatment option against malaria in many countries following the widespread malaria parasite resistance to more affordable anti-malarial drugs such as chloroquine and sulphadoxine-pyrimethamine (SP) [2]. Furthermore, quinine remains the most widely used anti-malarial drug in the treatment of severe and complicated malaria in many malaria-endemic regions [3,4]. Kenya adopted the new malaria policy in 2004, which recommended the use of artemether 20 mg-lumefantrine 120 mg (AL) as the first-line drug for treatment of uncomplicated malaria [4]. Apart from AL, other artemisinin-based combinations and monotherapy drugs are widely available and easily accessed in private outlets in Kenya [5,6] and other malaria-endemic countries [7].

The implementation of a policy is a continuous process and involves many activities including, but not limited to, in-service training (to update the personnel in the field with new knowledge) and adoption of new practices (which comes along with changes in treatment policy). In Kenya, there is a greater effort in training of health service providers in the public sector on the use of ACT relative to the private sector despite the vital role they play in malaria treatment [7,8]. Data on evaluations of knowledge and practices before or during implementation of the ACT, and quinine as a second-line anti-malarial in the private sector in relation to the other sectors in malaria-endemic region of Kenya, is scanty [8-11]. In addition, the multiple-dose regimens for most ACT and quinine are rather more complicated in comparison to a single dose needed for SP. This situation has further been complicated by the fact that previous findings in Kenya demonstrate that only 11% of health workers dispensing AL in public facilities were without formal clinical qualification [8].

Studies on health-worker practices have reported mixed findings. For example, a previous study to determine the predictors of quality of health-worker practices reported failure of health workers to prescribe AL to all deserving cases due to insufficient supply of AL [11]. This observation further raised fears of stockouts and patients’ preferences for SP over AL because of its simple dosage [11]. In the same study, AL was provided free to consumers, however, the health workers had to assess and prioritize cases that they deemed deserved to receive AL as it was considered expensive by the government. Other practices included prescription of available drugs such as amodiaquine since they were continuously supplied to the health facilities despite the policy change to ACT [11].

The successful policy outcome in the appropriate use of ACT and quinine, therefore, is dependent on provision of suitable knowledge to the health providers in all drug outlets and to consumers. Currently, literature on providers’ knowledge and practices on treatment policy and dosing regimens on the use of AL following the policy change in Kenya is dwindling. Despite the use of quinine for decades, no study has been carried out to evaluate providers’ knowledge on its use for malaria treatment in Kenya. The current study evaluated providers’ knowledge and practices of treatment policy and dosing regimens with AL and quinine in the public, private and not-for-profit drug outlets.

## Methods

### Study design and study area

A cross-sectional survey was conducted between February and May 2012 in two regions of Nyanza Province that are considered endemic for *Plasmodium falciparum* transmission, but with different levels of risk for malaria. The regions are in the lowlands around Lake Victoria (Kisumu, Siaya and Bondo regions), which are experiencing a holo-endemic and stable *P. falciparum* transmission (altitude 0–1,300 m) and in the highlands of Kisii (Kisii, Gucha and Nyamira regions), which are experiencing an epidemic transmission (>1,300-1,750 m) [12].

### Sample size and sampling

A three-stage sampling approach was used to survey drug outlets. For each region, a list of public medical facilities was compiled using a database obtained from the Ministry of Health [13]. First, from each of the two selected survey areas, the main public hospital was selected, and then a further five hospitals under the main facility were randomly sampled. Lastly, eight other public outlets under each of the hospitals were surveyed, bringing a total of 96. A matching number of private outlets was randomly selected. All (66) not-for-profit outlets and an additional 30 public health facilities were sampled to reach the required sample size of 288. The sample size of 288 was determined according to previous WHO recommendations for this kind of studies [14]. An outlet in the context of the current study was a registered/licensed dispensing site or any point of sale or provision of anti-malarial drugs.

### Data collection

Data were collected through interview by enumerators who visited the outlets in pairs. Prior to data collection, enumerators who had nursing qualification were trained on the use of the tools, followed by pilot testing with regular supervision in nine of each of the outlet type (in the same study area but whose data were not included in the study samples). Drug providers were recruited to participate in the survey. Those who consented to participate were asked two screening questions to determine whether (1) the outlet had stocked AL and/or quinine within the previous three months, and (2) AL and/or quinine were available on the day of the survey. All providers answering “yes” to at least one of the questions were recruited and gave information about the AL and/or quinine after informed consent was obtained from them. In outlets with more than one attendant, the person serving the clients at the time of the survey provided the information. The information collected included the type of qualification, any in-service training in the past two years, knowledge of treatment policy, knowledge of dosing regimen with AL and quinine, and awareness of recently banned anti-malarials. Practices of providers when dispensing AL and quinine were evaluated. Practices evaluated included whether they request for written prescription, prescribe AL, sell partial packs and the advice they gave to clients when dispensing AL. Ethical clearance to conduct the study was provided by the Maseno University Ethical Review Committee.

### Data management and statistical analysis

Data collected were checked in the field and at the end of each day cleaned to ensure completeness, consistency, credibility and eligibility. Information captured in open-ended questionnaire to test provider knowledge of treatment policy and dosing regimen and the practices involved when dispensing AL and quinine from the three outlet types, was coded and entered into Statistical Package for Social Sciences (SPSS) (Version 19). For comparison, chi-square analyses were used to test differences in proportions, and logistic regression analysis was used to determine whether training in the ACT policy influenced knowledge and practices of the providers at the outlets. Statistical significance of all the analyses was assessed at a *P* ≤ 0.05.

## Results

### Providers’ type of health qualification

In the current study, a total of 288 drug providers were interviewed. Included were level 5 hospitals, district/sub-district hospitals, health centres and dispensaries. For each of these, a matching private and not-for-profit outlet was surveyed. When stratified into outlets, respondents dispensing anti-malarials on the day of the survey in public outlets were 126 (44.0%), private were 96 (33.0%) and not-for-profit outlets were 66 (23.0%). The qualification of the drug providers varied with the outlet type as shown in Table 1. Overall, the majority were nurses 105 (36.5%), with the other major group comprised of pharmacists 95 (33.0%). Counsellors were 23 (8.0%), midwives 16 (6.0%), laboratory technicians 9 (2.0%), and clinical officers, relatives and shop assistants each comprised of <5 (2.0%). Other training not mentioned in the questionnaire comprised of 30 (10.4%) and included community health workers, nurse aids and nutritionists, who were mainly dispensing in private and not-for-profit outlets. Additional results revealed that there were more nurses dispensing drugs in the public outlets (60.3%) than in the private (1%) and not-for-profit (42.4%) outlets, while pharmacists were commonly found in private (54.2%) relative to public (18.3%) and not-for-profit (30.3%) outlets (Table 1).

**Table 1:** Drug providers’ qualifications by the outlet type

**Health qualification**	**Public**	**Private**	**Not-for-profit**
	**n = 126**	**n = 96**	**n = 66**
**Pharmacist**	23 (18.3%)	52 (54.2%)	20 (30.3%)
**Midwife**	13 (10.3%)	-	3 (4.5%)
**Clinical officers**	4 (3.2%)	-	-
**Nurse**	76 (60.3%)	1 (1.0%)	28 (42.4%)
**Laboratory Technician**	-	3 (3.1%)	6 (9.0%)
**Counsellors**	6 (4.7%)	17 (13.5%)	-
**Shop Assistant**	-	4 (4.2%)	-
**Relative**	-	2 (2.7%)	-
**Others**	4 (3.2%)	17 (13.5%)	9 (13.6%)

Analyses performed by Chi-square tests. *Statistically significant at *P* ≤ 0.05.

### In-service training of providers

Table 2 presents the proportion of staff receiving in-service training by outlet type. Results revealed that less than half of the respondents 142 (49.3%) had received in-service training on the use of AL as a first line-treatment of malaria within the two years prior to the current study. The training was mainly performed by non-governmental organizations (NGOs) 102 (72%) (mainly Global Fund), although government personnel offered health briefs regularly in the public facilities. The proportion of those who have received training within the previous two years were significantly higher in the public 91 (72.2%) relative to the private outlets 15 (15.6%; *P <* 0.0001; Table 2).

**Table 2:** Training of staff by outlet type

**Training within last two years**	**Public**	**Private**	**Not-for-profit**	***P*-value**
	**n = 126**	**n = 96**	**n = 66**	
**Yes**	91 (72.2%)	15 (15.6%)	36 (54.5%)	<0.0001*
**No**	20 (15.9%)	74 (77.0%)	27 (40.9%)	
**Do not know**	16 (12.7%)	7 (7.3%)	3 (4.5%)	

Analyses performed by Chi-square tests. *Statistically significant at *P* ≤ 0.05.

### Provider knowledge of treatment policy with artemether-lumefantrine and quinine

In order to establish the provider’s knowledge on treatment policy with AL and quinine, the distribution of proportions of providers’ knowledge against drug outlets was performed. Results revealed that the proportion of providers with knowledge of treatment policy were at its maximum in public outlets 126 (100.0%) and almost all 65 (98.4%) in not-for-profit relative to private 49 (51.0%). These individuals were able to mention AL as first line-treatment as recommended by the government for uncomplicated malaria. Similarly, those who were able to name quinine correctly as treatment for severe malaria and as a second-line anti-malarial were 121 (96.0%) in public, 55 (83.3%) in not-for-profit and 46 (47.9%) in private outlets. The proportions of those who knew that AL is a first-line anti-malarial were significantly higher in the public 126 (100.0%) and not-for-profit 65 (98.4%) relative to private outlets 49 (51.0%; *P* < 0.0001) (Table 3).

**Table 3:** Provider knowledge of treatment policy by outlet type

**Treatment policy**		**Public**	**Private**	**Not-for-profit**	***P-*value**
		**n = 126**	**n = 96**	**n = 66**	
**First-line treatment with AL**	Correctly	126 (100.0%)	49 (51.0%)	65 (98.4%)	<0.0001*
	Incorrectly	0 (0.00%)	47 (49.0%)	1 (1.6%)	
**Second-line treatment with quinine**	Correctly	121 (96.0%)	46 (47.9%)	55 (83.3)%	<0.0001*
	Incorrectly	5 (4.0%)	50 (52.1)	11 (16.7%)	

Analyses performed by Chi-square tests. *Statistically significant at *P* ≤ 0.05.

### Provider knowledge of dosing regimens with artemether-lumefantrine and quinine

In order to establish providers’ knowledge on dosing regimen with AL and quinine, the distributions of proportions of providers’ knowledge on dosing regimen against drug outlets was performed. Table 4 shows the proportion of providers in public outlets that were able to state the correct AL and quinine doses in comparison with providers in private and not-for-profit settings. As shown, the level of knowledge on AL (*P <* 0.0001) and quinine (*P <* 0.0001) significantly differed across the outlets. Results further demonstrated significantly higher proportions of providers’ correct knowledge at public outlets 119 (94.4%) on AL doses in children weighing 9 kg (*P* < 0.0001) relative to private 75 (78.1%) and not-for-profit 58 (78.8%). Of the participants in public outlets, a significantly (*P <* 0.0001) higher proportion 116 (92.0%), were able to state correctly the recommended doses with AL for adults weighing 45 kg as compared with 55 (57.3%) and 58 (78.8%) in private and not-for-profit outlets, respectively (Table 4).

**Table 4:** Provider knowledge of dosing regimens of artemether-lumefantrine and quinine by outlet type

		**Public**	**Private**	**Not-for-profit**	***P***-**value**
		**n = 126**	**n = 96**	**n = 66**	
**Children 9 kg**	Correct AL dose	119 (94.4%)	75 (78.1%)	58 (78.8%)	<0.0001*
	Incorrect AL dose	7 (5.6%)	21(21.9%)	8 (21.2%)	
	Correct quinine dose	111 (88.1%)	50 (52.1%)	37 (56.0%)	<0.0001*
	Incorrect quinine dose	15 (11.9%)	46 (47.9%)	29 (44.0%)	
**Adults 45 kg**	Correct AL dose	116 (92.0%)	55 (57.3%)	58 (78.8%)	<0.0001*
	Incorrect AL dose	10 (8.0%)	41 (42.7%)	8 (21.2%)	
	Correct quinine dose	112 (88.8%)	31 (32.3%)	43 (65.2%)	<0.0001*
	Incorrect quinine dose	14 (11.2%)	65 (67.7%)	23 (34.8%)	

Analyses performed by Chi-square tests. *Statistically significant at *P* ≤ 0.05.

### Provider awareness on currently banned drugs

When inquiries were made about recent government bans on anti-malarials, more than half 164 (56.9%) of all providers reported that they were aware of the most recent government ban on some anti-malarials and correctly observed that SP recommendations changed from treatment for mild malaria to Intermittent Preventive Treatment in Pregnancy (IpTp) in high risk areas. The reason for this change was identified as malaria drug resistance by 108 (37.5%) providers, although 83 (28.8%) could not give any reason, while 97 (33.7%) gave other reasons. Table 5 shows the provider awareness on recent government ban by outlet type.

**Table 5:** Provider awareness on currently banned drugs by outlet type

**Awareness on currently banned anti-malarials**	**Public**	**Private**	**Not-for-profit**	***P*-value**
	**n = 126**	**n = 96**	**n = 66**	
**Correctly naming SP**	108 (85.7%)	29 (30.2%)	27 (41.0%)	<0.0001*
**Naming other drugs**	15 (11.9%)	43 (44.8%)	27 (41.0%)	
**Not aware about the ban**	3 (2.4%)	24 (25.0%)	12 (18.0%)	

Analyses performed by Chi-square tests. *Statistically significant at *P* ≤ 0.05.

### Associations between in-service training and knowledge on treatment policy and drug regimen on artemether-lumefantrine and quinine

Additional logistic regression analysis was performed to identify knowledge variables that were associated with in-service training of staff. Results revealed that correct mentioning of drug recommended by government for treatment of uncomplicated malaria (OR; 1.07, 95% CI, 1.03-2.44, *P* = 0.040), treatment regimen for uncomplicated malaria in children (OR; 2.01, 95% CI, 1.66-3.83, *P* = 0.039) and in adults (OR; 2.03, 95% CI, 1.68-3.80, *P* = 0.039), and treatment regimen for severe malaria in children (OR; 2.66, 95% CI, 1.88-5.44, *P* < 0.0001) and in adults (OR; 2.01, 95% CI, 1.88-4.25, *P* = 0.002) was positively influenced by in-service training of the providers (Table 6).

**Table 6:** Influence of in-service training on knowledge factors

	**OR**	**95% CI**	***P*-value**
**Drug recommended for treatment of uncomplicated malaria**	1.07	1.03-2.44	**0.040**
**Treatment regimen for uncomplicated malaria in a child weighing 9 kg**	2.01	1.66-3.83	**0.039**
**Treatment regimen for uncomplicated malaria in an adult weighing 45 kg**	2.03	1.68-3.80	**0.039**
**Drug for treatment of severe malaria**	1.44	0.78-1.99	0.142
**Treatment regimen for severe malaria in a child weighing 9 kg**	2.66	1.88-5.44	**<0.0001**
**Treatment regimen for severe malaria in an adult weighing 45 kg**	2.01	1.88-4.25	**0.002**
**Awareness of recent ban**	1.51	0.65-2.01	0.158

Logistic regression analysis performed between dependent and independent variables to identify knowledge variables significantly associated with in-service training of staff. The *P*-values in bold were statistically significant at *P* ≤ 0.05; OR = Odd Ratios. 95% CI = 95% Confidence Interval.

### Provider practices on ACT

Further inquiries on the provider practices when dispensing ACT was carried out. Results showed that providers who requested for written prescription when dispensing AL was 109 (86.3%) in public, 22 (22.8%) in private, and 52 (79.1%) in not-for-profit outlets. Providers who did prescribe AL to their customers were 86 (67.9%) in public, 13 (37.6%) in private and 19 (29.1%) in not-for-profit outlets (Table 7). Further results demonstrated that 79 (82.3%) providers in the private outlets sell partial packs of AL, while only 50 (39.7%) of providers in public outlets advise taking first dose immediately and stress on the need for adherence to timing 9 (7.1%). None of the providers mentioned the need to take AL with a lot of fluids (Table 7).

**Table 7:** Provider practices on ACT by outlet type

		**Public**	**Private**	**Not-for-profit**
		**(n = 126)**	**(n = 96)**	**(n = 66)**
**Request for written prescription**	Yes	109 (86.3%)	22 (22.8%)	52 (79.1%)
	No	17 (13.7%)	74 (77.2%)	14 (21.2%)
**Prescribes AL**	Yes	86 (67.9%)	13 (37.6%)	19 (29.1%)
	No	40 (31.6%)	83 (72.4%)	47 (70.9%)
**Sells partial packs of AL**	Yes	1 (1.0%)	79 (82.3%)	8 (12.1%)
	No	125 (99.0%)	17 (17.7%)	58 (87.9%)
**Advice given when dispensing AL**				
**Take first dose immediately**		50 (39.7%)	8 (8.0%)	7 (11.2%)
**Need for adherence to timing**		9 (7.1%)	21 (21.8%)	53 (81.1%)
**Verbal description of treatment course**		1(1.0%)	5 (5.2%)	1 (1.0%)
**Side effects**		11 (8.7%)	10 (10.4%)	-
**Drug interactions**		-	-	1 (1.0%)
**Importance of adherence to treatment course**		45 (35.7%)	3 (3.0%)	3 (4.9%)
**Taking with a lot of fluids**		0 (0.0%)	0 (0.0%)	0 (0.0%)
**Others**		2 (1.6%)	15 (15.6%)	-
**None given**		7 (5.6%)	35 (36.5%)	1 (1.9%)

Distributions of proportions performed by Chi-square tests.

Further logistic regression analyses demonstrated that request for written prescription (OR; 3.00, 95% CI, 2.45-10.4, *P* = 0.001), prescription of AL (OR; 4.03, 95% CI, 2.99-14.7, *P* < 0.0001) and selling of partial packs (OR; 3.79, 95% CI, 2.77-11.2, *P* < 0.0001) were positively influenced by the in-service training of the provider (Table 8).

**Table 8:** Influence of in-service training on provider practices

**PProvider’s practices**	**OR**	**95% CI**	***P-*value**
Request for written prescription	3.00	2.45-10.4	**0.001**
Prescription of AL	4.03	2.99-14.7	**<0.0001**
Selling partial packs of AL	3.79	2.77-11.2	**<0.0001**
Advice given when dispensing AL	1.5	0.77-2.06	0.208

Logistic regression analysis between independent and dependent variables was used to identify practices that were associated with in-service training of providers. The P-values in bold were statistically significant at *P* ≤ 0.05; OR = Odd Ratios. 95% CI = 95% Confidence Interval.

## Discussion

This survey was performed six years after the government of Kenya had adopted the policy on ACT for treatment of malaria. This policy recommended the use of AL as a first-line treatment for uncomplicated malaria, while quinine was still preferred for treatment of complicated and severe malaria. In 2010, the policy was universally changed to include the use of ACT for only laboratory-confirmed malaria cases [15,16]. The current study therefore compared knowledge and practices in providers in public, private, and not-for-profit outlets on treatment policy and dosing regimen on recommended anti-malarials in malaria-prone areas of western Kenya.

The results revealed that there were more in-service trained personnel in the past two years dispensing drugs in public outlets (72.2%). This figure surpassed the target 60% set up by the Ministry of Health (MoH) in 2006 [17] and was much higher than the figure (46%) previously reported in another study conducted one year after the policy change in Kenya [8]. However, this proportion trained is below a recent proposed target of 100% as per the Division of Malaria Control (DOMC) Monitoring and Evaluation report for 2013 [18]. The expansion of the in-service training coverage in public facilities is encouraging despite the fact that the private sector is still lagging behind in training its drug providers. Further findings demonstrated that a considerable bulk (45%) of private outlet staff dispensing drugs had no clinical qualification. This confirms previous findings in Kenya and Democratic Republic of Congo [19,20] in which it was shown that a lower proportion of drug providers at the private outlets were trained. The private-sector qualification has been a staggering issue for a very long time in developing countries despite the significant role they play in medication management and provision of relevant information to patients [21]. The findings in the private sector were contrary to that in the not-for-profit outlets in that a higher proportion of drug providers (54.5%) were trained in the not-for-profit outlets. This disparity in the proportion of those trained in private *vs* not-for-profit outlets may be attributed to the fact that the mission hospitals (which are the major outlets in the not-for-profit category), receive government support and tend to effect policies enacted by the government. The 54.5% training received in this sector is nevertheless inadequate and may have been contributed by the recent mushrooming of non-governmental interventions in Nyanza Province (e.g. The Millenium Villages Projects and Ogra Foundation), most of which targets integrated management of HIV and AIDS and malaria. The qualification of providers in this sub-sector has not yet been evaluated. Meanwhile, for better results, both the private and all players in the not-for-profit sector need to be involved in the implementation of ACT policy. In addition, the providers should have regular and adequate training in drug dispensation to ensure correct administration of anti-malarials.

An interesting observation was that about half (49%) of providers in private outlets could not mention AL as first-line treatment for uncomplicated malaria, while approximately 52% mentioned quinine incorrectly for treatment of severe malaria. This observation provides additional evidence of inadequate knowledge and skills on the treatment policy. The reason behind low knowledge of quinine could be attributed to it being less frequently stocked by the private sector due to reasons, such as: quinine is mostly prescribed as a second-line drug and therefore most customers would only ask for it after the failure of other drugs, or it is either less publicized or not publicized at all in media, and there is decreasing awareness about it, as compared to first-line AL. Private providers tend to stock drugs that are frequently sought by consumers as it was demonstrated in a previous study that the type of drug acquired for use in a particular region is influenced by awareness on the type of anti-malarial in the market [22].

Questions were further raised on the dosing regimen with AL and quinine for particular weights (children weighing 9 kg and adults weighing 45 kg) for uniformity and easy recording. Disparity was recorded in knowledge of dosing regimen with the two anti-malarials in public, private and not-for-profit outlets. For example, a generally higher knowledge of dosing regimen for both children and adults was observed in public and not-for-profit outlets. This was in part influenced by in-service training by non-governmental bodies reported in this study, an indication of need for training. Some studies related to prescription of AL reported that incorrect weight-specific prescriptions of AL were sporadic and the packaging would have influenced dosing regimen [23]. One previous study carried out in Tanzania concurs with the current findings in that most dispensers in private pharmacies could not state the dosing schedules of AL without referring to the package leaflets [24]. A higher knowledge of treatment regimen with quinine for severe malaria in public outlets reported in the current study could have potentially been influenced by the concurrent in-service training and health briefs, contrary to previous observations in Democratic Republic of Congo, in which it was shown that training of the providers did not improve the knowledge of the dosing schedule with quinine among the Village Health Volunteers (VHV) and pharmacy owners [25]. However, the reasons for the differences on awareness level on the most recent ban on anti-malarials being observed in the outlets still remains unclear.

In the private and not-for-profit outlets, the providers knew more than three symptoms of malaria, a good indication for subsequent prescription, even though the malaria treatment guidelines still confine AL and quinine prescription to registered pharmacies and should only be provided to confirmed cases of malaria. It is important to mention that although the guidelines insist on laboratory tests before selling the policy-recommended drugs to customers, the practice of providers in private outlets selling anti-malarials without prescription has been observed in various outlets in the study region and this practice was significantly influenced by in-service training.

The providers at the drug outlets are charged with the responsibility of advising their customers on all matters pertaining to adherence to treatment schedule and proper usage of drugs. These practices are to be enhanced by the providers since the policy indicates that there should be inclusion of a written prescription, sale of full doses of drugs, and even advice to take the first dose immediately. In addition, the providers should educate the patients on the need for adherence, the possible side effects and the need to take more fluids during the treatment. It is encouraged that the providers verbally describe the treatment course and the importance of adherence.

The discrepancy in the practices by the providers in private outlets is disquieting. The sale of partial packs of AL shows lack of commitment to change even in the face of ACT, despite the fact that the type and duration of treatment in private outlets is determined by the clients’ ability to pay [26,27]. The low prescription of AL in private and not-for-profit outlets could be attributed to lack of or insufficient in-service training. This followed the observation that training influenced the prescription practice with better prescription seen in the public sector (67.9%). In a previous study exploring reasons for health workers not prescribing AL despite the drug being in stock at the public health facilities, it was observed that most of the health workers were only responding to general health system weaknesses leading to non-adherence to the treatment guidelines [11]. A low level of prescriptions of the nationally recommended drug was reported in yet another study where only 26% of children who needed treatment with AL received a prescription for this drug according to national guidelines [23]. The deviation from the guidelines observed in the current study shows inadequate skills and knowledge in preparation for implementation of the new policy in the private sector.

It is important to note that the difference in the outcome observed in the current study is attributed to in-service training and health briefs, which in essence should take precedence in readiness for the on-going implementation process. There is critical need to find an intervention which would address this disparity, owing to the importance of this sector in health provision and given that absence of training in AL policy influence drug-prescribing practices in Kenya [28]. One way of increasing coverage can be through training the trainers from the same sector who will further train their counterparts. This has been shown to improve malaria treatment in private drug outlets in Bungoma District in western Kenya prior to policy change [29]. Another approach may be to train the drug dispensers directly in organized workshops, give information, sensitization and education on the new policy and health briefs on quinine regimen, although this method might be a more expensive endeavour for the government. A previous study reported improvement on health-worker performance in care and treatment of patients after conducting educative seminars and training of health workers [30], confirming a hypothesis of increased training and awareness prior to implementation of a change in drug policy.

Focus on public sector to implement policy change was reported in a study previously carried out in Kenya a year after the implementation process of the new malaria policy [8]. In that study health workers were trained in a cascade manner leading to an increased number of trained providers [8]. The tendency to focus on public sector to implement changes in new policy guidelines downplays the call by the World Health Organization to include private sector in malaria treatment due to their significant role in pharmaceutical management and provision of relevant information to patients, thus enhancing the improvement of rational drug use [21]. Formulation of good policies may not be a guarantee for proper interpretation of knowledge and practice. It must be accompanied by sensitization for the achievement of the outcome. A recent study carried out in neighbouring Tanzania reported positive results in achieving policy change through sensitization of targeted communities prior to the implementation process [31].

The current study had limitations in that it was only a one-time point survey and thus it could not evaluate the impact of other factors on provider’s knowledge and practices on dispensation of antimalarial drugs. Furthermore, the current findings are based on a sample of 288 outlets. As such, it would be critical to carry out an extensive longitudinal survey in a wider coverage of the outlets in the endemic areas to exhaustively delineate the impact of other factors on provider’s knowledge and practices on dispensation of anti-malarial drugs.

## Conclusion

Public-sector providers have better knowledge and better practices on treatment policy and dosing regimen with policy-recommended anti-malarials than their counterparts in private and not-for-profit sectors. Changes in treatment guidelines should be accompanied by subsequent implementation activities, which should involve all sector players. The assumption that providers will follow the guidelines on paper is merely a guess and therefore there is need for frequent sensitization through education, information and communication. Furthermore, monitoring and evaluation for the achievements and failures of the policy changes and subsequent re-planning for better results is compulsory. This will increase rational use of anti-malarials and reduce parasitic resistance to recommended drugs.

## Competing interests

The authors declare that they have no competing interests.

## Authors’ contributions

CAW designed, carried out the survey studies in the drug outlets and participated in the drafting of the manuscript. JHO, ROO, BOA and CO participated in the drafting of the manuscript. CAW and CO performed the statistical analysis. All authors read and approved the final manuscript.
